# Evidence-based clinical practice guidelines for cholelithiasis 2021

**DOI:** 10.1007/s00535-023-02014-6

**Published:** 2023-07-15

**Authors:** Naotaka Fujita, Ichiro Yasuda, Itaru Endo, Hiroyuki Isayama, Takuji Iwashita, Toshiharu Ueki, Kenichiro Uemura, Akiko Umezawa, Akio Katanuma, Yu Katayose, Yutaka Suzuki, Junichi Shoda, Toshio Tsuyuguchi, Toshifumi Wakai, Kazuo Inui, Michiaki Unno, Yoshifumi Takeyama, Takao Itoi, Kazuhiko Koike, Satoshi Mochida

**Affiliations:** 1 Guidelines Committee for Creating and Evaluating the “Evidence-Based Clinical Practice Guidelines for Cholelithiasis’’, The Japanese Society of Gastroenterology, 6F Shimbashi i-MARK Building, 2-6-2 Shimbashi, Minato-ku, Tokyo, 105-0004 Japan; 2 Miyagi Medical Check-up Plaza, 1-6-9 Oroshi-machi, Wakabayashi-ku, Sendai, Miyagi 984-0015 Japan

**Keywords:** Cholelithiasis, Gallbladder stones, Common bile duct stones, Intrahepatic stones, Endoscopic treatment, Surgical treatment

## Abstract

The Japanese Society of Gastroenterology first published evidence-based clinical practice guidelines for cholelithiasis in 2010, followed by a revision in 2016. Currently, the revised third edition was published to reflect recent evidence on the diagnosis, treatment, and prognosis of cholelithiasis conforming to the Grading of Recommendations Assessment, Development, and Evaluation (GRADE) system. Following this revision, the present English version of the guidelines was updated and published herein. The clinical questions (CQ) in the previous version were reviewed and rearranged into three newly divided categories: background questions (BQ) dealing with basic background knowledge, CQ, and future research questions (FRQ), which refer to issues that require further accumulation of evidence. Finally, 52 questions (29 BQs, 19 CQs, and 4 FRQs) were adopted to cover the epidemiology, pathogenesis, diagnosis, treatment, complications, and prognosis. Based on a literature search using MEDLINE, Cochrane Library, and Igaku Chuo Zasshi databases for the period between 1983 and August 2019, along with a manual search of new information reported over the past 5 years, the level of evidence was evaluated for each CQ. The strengths of recommendations were determined using the Delphi method by the committee members considering the body of evidence, including benefits and harms, patient preference, and cost–benefit balance. A comprehensive flowchart was prepared for the diagnosis and treatment of gallbladder stones, common bile duct stones, and intrahepatic stones, respectively. The current revised guidelines are expected to be of great assistance to gastroenterologists and general physicians in making decisions on contemporary clinical management for cholelithiasis patients.

## Introduction

Since the decision made by the Japanese Society of Gastroenterology (JSGE) in 2005 to publish evidence-based guidelines for clinically significant common gastrointestinal disorder, 11 clinical practice guidelines have been published. Cholelithiasis was selected as one of the target diseases, and the first edition of “Clinical practice guidelines for the treatment of cholelithiasis” was published in 2009, based on a literature search using the Igaku Chuo Zasshi, PubMed, and Cochrane library from 1983 to 2007. Some of the recommendations and statements, however, were determined by referring to old data or expert opinions due to the lack of available evidence. In 2016, its revised second edition, titled “Evidence-based clinical practice guidelines for the treatment of cholelithiasis”, was published with the cooperation of the Japan Gastroenterological Endoscopy Society and Japan Biliary Association, including new evidence published between 2007 and June 2012 [1].

Currently, following the JSGE policy of revising the guidelines every 5 years as necessary, the third revision has been issued to cover new insights after the publication of the second edition. This revision was carried out following the Minds Manual for Clinical Practice Development [2].

The committee of clinical practice guidelines for cholelithiasis consists of two subcommittees, namely, the development and evaluation committees, with refined new members. The former prepared and refined the draft and determined the final version by taking the suggestions of the latter into consideration.

In the current edition, the items to deal with are divided into three categories: clinical questions (CQs), core CQs, background questions (BQs), knowledge or practice that has been widely accepted, and future research questions (FRQs) which are questions that lack robust evidence at present and require further research to address a recommendation. Through discussions among committee members, eventually, 52 questions (29 BQs, 19 CQs, and 4 FRQs) were adopted covering the epidemiology, pathogenesis, diagnosis, treatments, complications, and prognosis.

A literature search was performed in the same manner as in the second revision, extending the search period to August 2019. A supplemental manual search was also carried out by each committee member, and some latest articles published after the above-mentioned period were also included in the reference as necessary. Structured abstracts were prepared for the CQs and FRQs, and the quality of evidence for each article was ranked as follows: A, high; B, moderate; C, low; and D, very low. [3]

The strengths of recommendations were determined by assessing the body of evidence using not only the data-driven approach but also patient preferences and cost–benefit balance according to voting by the committee members. Consensus was defined in advance as the acquisition of votes 70% or over [4], and the consensus rate was shown following each recommendation.

The present article summarizes the new guidelines to support gastroenterologists and general physicians in decision-making on the clinical management of cholelithiasis patients providing comprehensive statements for BQs and commentary, along with recommendations/statements for CQs and FRQ.

## Epidemiology and pathophysiology

### BQ1-1. Is the prevalence of cholelithiasis increasing in Japan?

#### Statement

There are no data showing recent trends in the prevalence of cholelithiasis in Japan. However, it is assumed to have increased with the increasing obese population, which is a risk factor for cholelithiasis.

– The incidence of intrahepatic stones has decreased in recent years but has been increasing again with the increase in biliary surgery.

These statements were made with reference to the following information and papers [5–11].

## BQ1-2. What are the causes of cholecystolithiasis?

### Statement

- The formation of cholesterol gallstones is associated with the supersaturation of cholesterol in bile, crystallization, and decreased contractility of the gallbladder. Such conditions can be caused by excessive calorie and animal fat intake, hyperlipidemia, oral contraceptive use, prolonged fasting, dieting, decreased intestinal motility, obesity, and so on.
- The main cause of calcium bilirubinate stones is biliary infection. Cholestasis is also associated with the formation.
- The formation of black stones is presumed to be due, in part, to an increase in unconjugated bilirubin in bile owing to an excessive supply of bilirubin. Such a condition can be caused by hemolytic diseases such as hereditary spherocytosis and thalassemia, Crohn's disease, and ileal resection. Stones of this kind are also seen in liver cirrhosis.

These statements were made with reference to the following information and papers [12–19].

## BQ1-3. What are the risk factors for gallbladder stones?

### Statement

- Forty (years of age), female, fatty, fair, and fertile (so-called “5 Fs”) remain strong risk factors. In addition, dyslipidemia, previous upper gastrointestinal surgery and bariatric surgery, and diet are also considered to increase the risk of gallbladder stones.

These statements were made with reference to the following information and paper [15, 20–48]

## BQ1-4. What is the natural history of gallbladder stones?

### Statement

- Approximately 70% of patients with gallbladder stone disease are found asymptomatic and often remain asymptomatic. In asymptomatic gallstones, the cumulative symptomatic rate over a 10 year period is estimated to be about 10–20%. In addition, there have occasionally been reports of cases in which biliary calculi have disappeared asymptomatically. Since obesity and lifestyle-related diseases are risk factors for gallbladder stones, higher cumulative mortality from cardiovascular disease, cancer, and diabetes has been reported in patients with gallbladder stones.

This statement was made with reference to the following information and papers [49–59].

## CQ1-1. Is gallbladder stone a risk factor for gallbladder cancer?

### Statement

- There is no clear evidence that gallbladder stones are a risk factor for gallbladder cancer.

(Recommendation: none, 100% agreed, evidence level D).

## Commentary

It is well known that patients with gallbladder cancer have gallbladder stones at a high rate (69–96%) [60]. Many case–control studies have been reported on the association between gallbladder stones and gallbladder cancer [61–76]. Although the relative risks vary from 2.3 to 34.4, gallbladder stones have been reported to be a risk factor for gallbladder cancer. In particular, patients with large stones [61] [76], numerous stones [71] [73], non-cholesterol stones (mixed stones), symptoms [70], and a long symptomatic period [71] reportedly have a high risk of gallbladder cancer. Three previous cohort studies showed a higher incidence of gallbladder cancer in patients with gallbladder stones than in normal controls (relative risk, 3.01–6.1) with a median follow-up period of 6.1–13.3 years [77–79]. A meta-analysis of three cohort studies and seven case–control studies also showed that gallstones were the strongest risk factor for gallstone cancer, with a relative risk of 4.9 [80]. In contrast, a large cohort study of 113,394 patients with a history of gallbladder stones/cholecystitis who were followed-up for 11 years showed a hazard ratio of 1.07, which suggests a negative impact of gallbladder stones on the occurrence of gallbladder cancer [81]. Based on these results, there is no dispute regarding the relationship, in some way, between gallbladder stones and gallbladder cancer. However, it is still difficult to determine whether gallbladder stones are the cause of gallbladder cancer, or whether they are more likely to form during the development of gallbladder cancer because the incidence of gallbladder cancer in patients with gallbladder stones is extremely low, and there is also controversial negative evidence.

Porcelain gallbladder and atrophic gallbladder have been considered high-risk factors for gallbladder cancer. However, a recent systematic review revealed that their roles as risk factors were not as significant as previously suggested [82–85].

## BQ1-5. Is hepatolithiasis a risk factor for intrahepatic cholangiocarcinoma?

### Statement

- Hepatolithiasis is a strong risk factor for intrahepatic cholangiocarcinomas.

This statement was made with reference to the following information and papers [86–116].

## Diagnosis

**Fig. 1:**
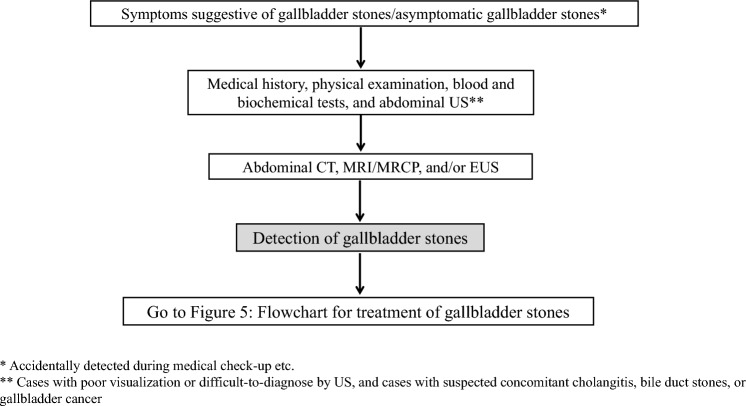
Flowchart for diagnosis of gallbladder stones

*Gallbladder stones* (*see *Fig. 1).

## BQ2-(1)-1. What are the symptoms of gallbladder stones?

### Statement

- Most cases of gallbladder stones are asymptomatic; however, characteristic abdominal pain, nausea, and vomiting may occur during an attack. Fever is also observed in cases of associated acute cholecystitis.

This statement was made with reference to the following information and papers [56, 117–120].

## BQ2-(1)-2. How should the diagnosis of gallbladder stones be made?

### Statement

- Abdominal ultrasound (US) and blood tests are performed in cases with symptoms suggestive of gallbladder stones. In cases of poor visualization of the gallbladder or indeterminate US findings, suspected cholangitis/bile duct stones, Mirizzi syndrome, confluence stones, or concomitant gallbladder cancer, abdominal computed tomography (CT), magnetic resonance imaging (MRI)/magnetic resonance cholangiopancreatography (MRCP), and/or endoscopic ultrasonography (EUS) are recommended. (Fig. 1)

This statement was made with reference to the following information and papers [121–125].

## BQ2-(1)-3. What about the diagnostic process of a patient with acute cholecystitis?

### Statement

**Fig. 2:**
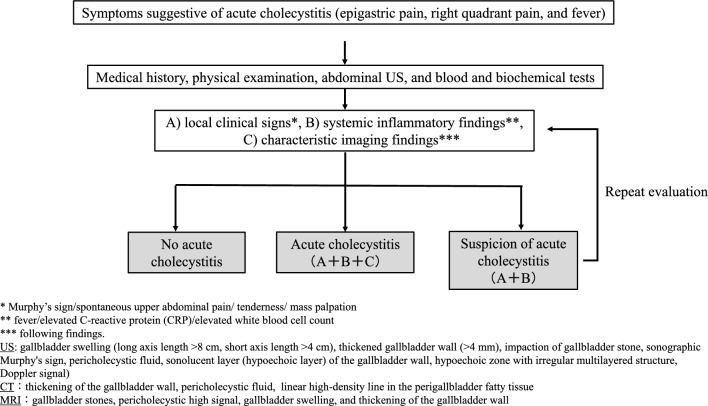
Flowchart for diagnosis of acute cholecystitis

- The diagnosis of acute cholecystitis is based on local clinical signs, systemic inflammatory findings, and diagnostic imaging. Severity is assessed based on organ dysfunction and local inflammatory findings. (Fig. 2)

This statement was made with reference to the following information and papers [126–141].

#### ***Common bile duct stones (CBDS) (see*** Fig. 3*).*

**Fig. 3:**
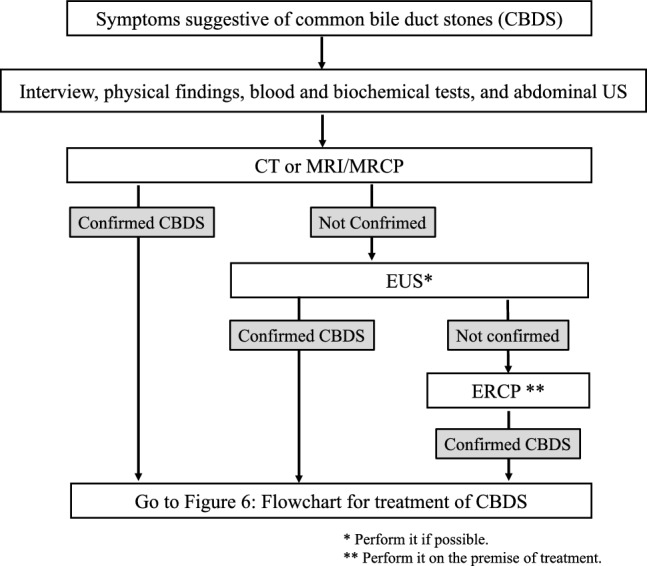
Flowchart for diagnosis of common bile duct stones

## BQ2-(2)-1. What are the symptoms of CBDS?

### Statement

- Patients with CBDS often present with symptoms such as abdominal pain, back pain, fever, jaundice, and nausea/vomiting but are sometimes asymptomatic.

These statements were made with reference to the following information and papers [10, 142, 143].

## BQ2-(2)-2. How are CBDS diagnosed?

### Statement

- In patients with suspected CBDS, abdominal US, abdominal CT, and/or MRI/MRCP are performed as appropriate. When these tests are indeterminate, EUS is suggested. Endoscopic retrograde cholangiopancreatography (ERCP) is recommended if symptoms relevant to acute cholangitis are present. (Fig. 3)

This statement was made based on the following information and papers [142, 144–153].

## CQ2-(2)-1. Is it recommended to perform EUS prior to ERCP rather than direct ERCP when abdominal US, CT, and/or MRI/MRCP are indeterminate in patients with suspected CBDS?

### Recommendation

- EUS prior to ERCP is suggested in patients with suspected CBDS when other diagnostic images are indeterminate.

(Recommendation: weak, 100% agreed, evidence level B).

## Commentary

EUS is known to have a high diagnostic capability for CBDS because of its high spatial resolution. According to a meta-analysis evaluating the diagnostic capability of EUS for CBDS, the sensitivity, and specificity were 0.94 (95% confidence interval [CI] 0.93–0.96) and 0.95 (95% CI 0.94–0.96), respectively [150]. Another meta-analysis compared the diagnostic capability of EUS and MRCP for CBDS, reporting that the sensitivities of EUS and MRCP were 0.97 (95% CI 0.91–0.99) and 0.87 (95% CI 0.80–0.93) (*p* = 0.006), specificities were 0.90 (95% CI 0.83–0.94) and 0.92 (95% CI 0.87–0.96) (*p* = 0.42), and the diagnostic odds ratios were 162.5 (95% CI 54.0–489.3) and 79.0 (95% CI 23.8–262.2) (*p* = 0.008), respectively [154]. Both EUS and MRCP showed a high diagnostic capability for CBDS, but the diagnostic odds ratio was significantly superior with EUS, which was considered to be due to the high sensitivity of EUS in detecting small stones [154]. EUS is also useful in diagnosing CBDS in patients with suspected biliary pancreatitis [155, 156].

A meta-analysis of four randomized controlled trials (RCTs) compared the efficacy of performing EUS prior to ERCP (EUS-first strategy) with direct ERCP (ERCP-first strategy) in patients with suspected CBDS [157], reporting that the EUS-first strategy could omit ERCP in 67.1% of the patients; however, the number of procedures in the EUS-first strategy was significantly higher than that in the ERCP-first approach (relative risk [RR] 2.46, 95% CI 1.34–4.52; *p* = 0.004) since ERCP was performed after EUS. As for safety, the EUS-first strategy significantly reduced the risks of overall adverse events (RR 0.35, 95% CI 0.20–0.62) and post-procedure pancreatitis (RR 0.21, 95% CI 0.06–0.83). EUS prior to ERCP in patients with suspected CBDS allows for omitting unnecessary ERCP and reduces the risk of adverse events. However, the limited availability of EUS is a problem because of the necessity of an experienced endosonographer.

### Column 1: is it possible to differentiate between primary CBDS and secondary stones from the gallbladder?

CBDSs include primary and secondary stones migrating from the gallbladder. It is generally believed that calcium bilirubinate stones are formed in the common bile duct (CBD), while cholesterol and black stones are formed in the gallbladder. However, because stones formed in the gallbladder undergo further modification after migrating into the CBD [158], it is difficult to determine whether CBDSs are formed in the CBD or gallbladder in clinical practice. A study on the correlation between CBDS and CBD diameter showed that the diameter of the CBD was significantly larger in patients with primary stones than in those with secondary stones, but this has not been widely accepted because of the small number of cases in this study.

From a therapeutic point of view, the method of bile duct stone removal is independent of the stone type, whether endoscopic or surgical. It has been pointed out that endoscopic treatment even with small-incision endoscopic sphincterotomy (EST) or balloon dilation may impair papillary function to a certain degree, leading to possible increased retrograde infection from the duodenal papilla and recurrence of common bile duct stones [158, 159]. On the other hand, laparoscopic common bile duct exploration (LCBDE) does not impair papillary function and is considered appropriate, especially for younger patients due to the reduced risk of ascending cholangitis.

Stone diseases are benign, and long-term follow-up is necessary. Accumulation of data on the nature of removed stones and continued follow-up may facilitate making an adequate selection of treatment options.

These statements were made with reference to the following information and papers [158, 159].

#### ***Hepatolithiasis (see*** Fig. 4*).*

**Fig. 4:**
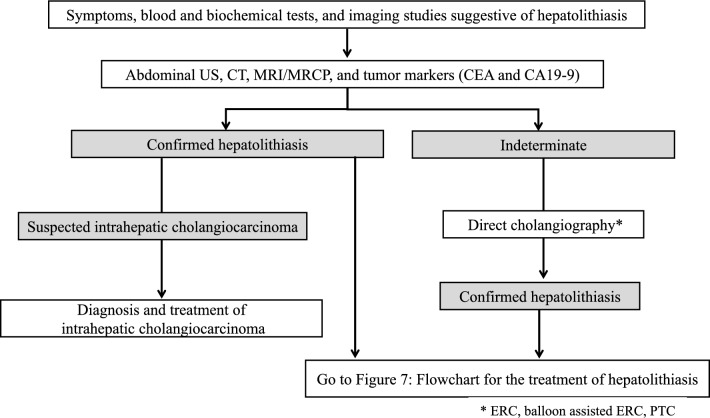
Flowchart for diagnosis of hepatolithiasis

## BQ2-(3)-1. What are the symptoms of hepatolithiasis?

### Statement

- Abdominal pain, fever, and jaundice are often observed in patients with hepatolithiasis; however, the number of asymptomatic patients has been increasing.

This statement was made with reference to the following information and papers [160–162].

## BQ2-(3)-2. How is hepatolithiasis diagnosed?

### Statements

– When hepatolithiasis is suspected, minimally invasive modalities, including blood examination, US, CT, MRI, and MRCP, are performed. Furthermore, measurement of tumor markers is recommended to assess the risk of intrahepatic cholangiocarcinoma.
– If intrahepatic stones are visualized and intrahepatic cholangiocarcinoma is suspected, a detailed examination to diagnose intrahepatic cholangiocarcinoma should be performed.
– In symptomatic cases or when intrahepatic stones cannot be visualized, direct cholangiography, bile duct cytology, and cholangioscopy are recommended, considering the comorbidity of intrahepatic cholangiocarcinoma.

These statements were made with reference to the following information and papers [11, 160, 161, 163, 164]. (Fig. 4).

## Treatment

**Fig. 5:**
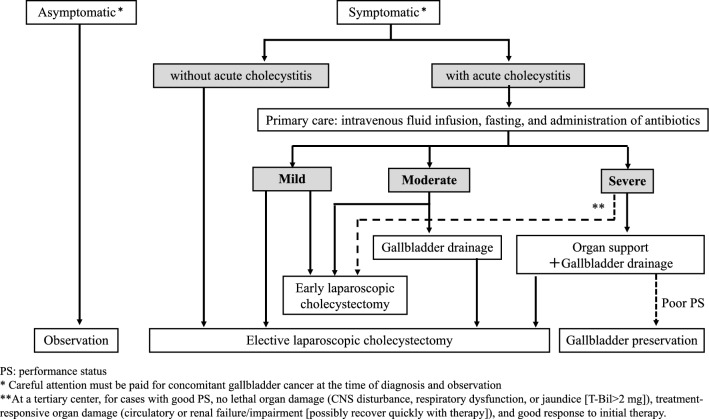
Flowchart for treatment of gallbladder stones

***Gallbladder stones (see*** Fig. 5*).*

## BQ3-(1)-1. How should the treatment of patients with acute cholecystitis be applied?

### Statement

- After initial treatment, such as adequate intravenous fluid infusion and administration of antibiotics and analgesics, further treatment should be performed based on the severity of acute cholecystitis.

This statement was made with reference to the following information and papers [165–173].

## BQ3-(1)-2. What are the nonsurgical treatments for cholecystolithiasis?

### Statements

- Butylscopolamine, flopropione, and nonsteroidal anti-inflammatory drugs are used to treat attacks.
- Oral ursodeoxycholic acid is an option for prophylaxis against attacks.
- Extracorporeal shock wave lithotripsy (ESWL) and oral chemical dissolution therapy have been used for the treatment of gallbladder stones but have rarely been performed in recent years.

These statements were made with reference to the following information and papers [119, 174–183].

## CQ3-(1)-1. Is cholecystectomy recommended over follow-up for asymptomatic patients with cholecystolithiasis?

### Recommendation

- Cholecystectomy should be considered for patients at high risk of gallbladder cancer.

(Recommendation: weak, 100% agreed, evidence level C).

## Commentary

Asymptomatic gallbladder stones are increasingly being detected due to the widespread use of medical examinations and advances in diagnostic imaging. It is estimated that 2–4% of asymptomatic gallbladder stones become symptomatic within 1 year of diagnosis [184, 185]. Two-thirds of asymptomatic patients are expected to remain asymptomatic for the remainder of their lives. Asymptomatic gallstones have a 0.3% chance of developing acute cholecystitis, 0.2% risk of leading to obstructive jaundice, and 0.04–1.5% risk of developing acute pancreatitis; however, Development of gallstone ileus is rare [186].

It is well known that the frequency of gallbladder cancer complicated by gallbladder calculus is as high as 40–70% [83]. However, the incidence of gallbladder cancer associated with cholelithiasis is approximately 0.1–0.5%, although the rate varies in available reports [85]. Furthermore, the incidence of gallbladder cancer from asymptomatic gallbladder stones is reported to be rare (0–0.5%) [187]. In addition, a decision analysis model showed that prophylactic cholecystectomy for patients with asymptomatic gallbladder stones did not improve their prognosis, including death from gallbladder cancer [188]. Generally, prophylactic cholecystectomy is in consideration of the possibility of developing gallbladder cancer and is not recommended for patients with asymptomatic gallbladder stones. However, cholecystectomy should be considered in patients at high risk of gallbladder cancer. The high-risk groups for gallbladder cancer include patients with stones larger than 3 cm, polyps larger than 10 mm, porcelain gallbladder, thickened gallbladder walls, and stone-filled gallbladders [50, 83, 85, 186, 189].

During follow-up, it is recommended to explain to the patients the risk of developing symptoms, acute cholecystitis, and gallbladder cancer, and the necessity to perform periodic examinations, such as abdominal US.

## FRQ 3-(1)-1. Is cholecystectomy recommended in a case of gallbladder stones with an atrophic gallbladder?

### Statement

- Cholecystectomy should be performed if gallbladder cancer is suspected based on the morphology of the thickened gallbladder wall and the course of the disease. However, it is difficult to distinguish and rule out the presence of gallbladder cancer from images; therefore, the indication for surgery in this condition should be established in future studies.

## Commentary

If no gallbladder is visualized or an atrophic gallbladder is found on imaging studies, it may be due to a hypoplastic gallbladder, chronic cholecystitis, or biliary gastrointestinal fistulas [190–192].

Chronic cholecystitis and biliary gastrointestinal fistulas are often associated with gallbladder stones, and it has been reported that 5.6% of the patients operated on for asymptomatic gallbladder stones have grossly atrophic gallbladders [193]. In biliary gastrointestinal fistulas, a sudden disappearance of symptoms associated with gallbladder stones (e.g., pain and jaundice) is often observed. In addition to stones, ulcers and malignant tumors of the gallbladder may cause fistulas. On imaging, an atrophic gallbladder is sometimes found to be associated with biliary emphysema [192].

The frequency of gallbladder cancer in patients with atrophic gallbladders is unknown. In a case series, 4.4% of the surgical cases of atrophic gallbladder were reported to have gallbladder cancer [194]. An atrophic gallbladder with stones has been considered an indication for cholecystectomy as a high-risk group for gallbladder cancer because the gallbladder wall is difficult to observe and evaluate entirely due to stones filling the lumen [83, 195]. In contrast, it has been suggested that an atrophic gallbladder is a condition in which the epithelium of the gallbladder has been abolished due to chronic cholecystitis; therefore, it is unlikely to become symptomatic or develop gallbladder cancer [85]. As it is difficult to diagnose or rule out the presence of gallbladder cancer from diagnostic imaging in atrophic gallbladders, cholecystectomy may be considered to rule out malignant tumors based on the degree of thickening of the gallbladder wall and the course of the disease. In general, a wall thickening of 3 mm is considered the upper limit of normal, but localized or diffuse irregular thickening is also included in the indication for surgery [196]. In addition, tumor markers and FDG-PET findings are used in clinical practice to determine the indications for surgery. Increased difficulty in surgery with an atrophic gallbladder or biliary gastrointestinal fistula should be kept in mind [197].

## CQ3-(1)-2. Is cholecystectomy recommended over nonoperative treatment for symptomatic gallbladder stones?

### Recommendation

- Laparoscopic cholecystectomy is recommended for symptomatic gallbladder stones.

(Recommendation: strong, 91% agreed, evidence level C).

## Commentary

Among patients with symptomatic gallbladder stones, 1–3% of the patients with mild symptoms develop severe symptoms or complications (i.e., acute cholecystitis/acute cholangitis, jaundice, and/or pancreatitis) within 1 year. The annual rate of patients with moderate symptoms who develop severe symptoms and undergo surgery is 6–8%, and this rate has decreased over time [55].

After nonoperative treatment of acute cholecystitis, emergency surgery due to flare-ups of symptoms while waiting for an elective cholecystectomy occurs in 6–23% of the cases [198]. In elderly patients, cholecystectomy is preferable if the patient's general condition is judged to be amenable to surgery because sudden recurrence is not uncommon, sometimes in more severe forms, after a long asymptomatic period. Laparoscopic cholecystectomy is a safe and useful treatment for symptomatic gallbladder stones in the absence of acute cholecystitis [199].

Complications associated with laparoscopic cholecystectomy include bile duct injury, bleeding, and injury to other organs. According to the results of a questionnaire survey by the Japanese Society for Endoscopic Surgery in 2017, bile duct injury, bleeding requiring laparotomy, and injury to other organs occurred in approximately 0.4%, 0.3%, and 0.3% of all laparoscopic cholecystectomies, respectively [200].

In a gastrointestinal quality of life index study of symptomatic patients with gallbladder stones, excluding those with acute cholecystitis, pancreatitis, and common bile duct stones, laparoscopic cholecystectomy was reported to significantly improve the quality of life of patients [201].

## CQ3-(1)-3. Is early laparoscopic cholecystectomy for severe acute cholecystitis recommended over conservative treatment, including biliary drainage and administration of antibacterial agents?

### Recommendation

Systemic treatment using antibacterial agents should be performed first in cases of severe cholecystitis.

- Early cholecystectomy by a skilled surgeon is recommended in a tertiary medical institution for patients who have undergone surgery.

(Recommendation: weak, 91% agreed, evidence level B).

- If a patient cannot withstand emergent/early surgery, early gallbladder drainage is recommended.

(Recommendation: strong, 100% agreed, evidence level A).

## Commentary

For severe acute cholecystitis with organ failure, systemic or local treatment alone is not recommended [202]. Biliary drainage or surgery should be performed to control local infection along with systemic treatments, such as the administration of antibacterial agents and supportive care for organ failure, depending on the severity of organ failure.

Early cholecystectomy is recommended if organ failure can be controlled. However, mortality is significantly higher in patients with severe acute cholecystitis and critical organ failure, including central nervous system disorder, respiratory dysfunction, and jaundice [203]. According to the Tokyo guidelines 2018, patients with mild-to-moderate cholecystitis, that is, those with an age-adjusted Charlson’s comorbidity index ≥ 6 points, and American Society of Anesthesiologists Physical Status score ≥ 3 points were included in the high-risk surgery group. Patients with severe acute cholecystitis should be treated at an institute with an intensive care unit. For surgical intervention, laparoscopic cholecystectomy should be performed by skilled endoscopic surgeons.

## CQ3-(1)-4. Is endoscopic gallbladder drainage recommended over percutaneous transhepatic gallbladder drainage in acute cholecystitis?

### Recommendations

- Percutaneous transhepatic gallbladder drainage (PTGBD) is recommended in cases of acute cholecystitis.

(Recommendation: strong, 100% agreed, evidence level B).

- Endoscopic gallbladder drainage is proposed for patients with coagulopathy, patients receiving antithrombotic agents, and those with ascites by a skilled endoscopist.

(Recommendation: weak, 100% agreed, evidence level B).

## Commentary

There are several methods for gallbladder drainage for acute cholecystitis, such as PTGBD, percutaneous transhepatic gallbladder aspiration, and endoscopic gallbladder drainage, including transpapillary gallbladder drainage (ETGBD) and EUS-guided gallbladder drainage (EUS-GBD). Table 1 presents the published clinical results for each drainage procedure. PTGBD is recommended for high-surgical risk patients because of its high success rate (technical success, 97–100%; clinical success, 89.3–97.6%) and its safety (complication rate, 3–39.5%) in previously published data and because of the simplicity of the technique [173, 204–214]. In contrast, ETGBD has been performed as an alternative treatment at skilled pancreatobiliary endoscopic institutions with a lower success rate (technical: 81–96%, clinical: 75–93%) than PTGBD [173, 211–214]. Therefore, ETGBD is considered the treatment of choice for patients with suspected CBDS, coagulation abnormalities, antithrombotic medications, ascites accumulation, and other conditions that make PTGBD difficult to perform, as well as for patients with a high risk of PTGBD-related complications to control acute cholecystitis. There have been few reports on the management of acute cholecystitis with coagulation abnormalities or anticoagulant use. Guidelines on interventional radiology allow the performance of PTGBD with single-agent aspirin when the risk of thromboembolism is high [215]. However, the Japanese DPC data reported that PTGBD is associated with a significantly higher risk of bleeding in patients with cholecystitis who are taking antithrombotic drugs. These data should be kept in mind, and a sufficient explanation about the risk of bleeding should be provided to the patient prior to performing PTGBD [216].

**Table 1:** Comparison of percutaneous and endoscopic-transpapillary drainages for cholecystitis

Author	Year	Study design	Procedures	Number of cases	Technical success (%)	Clinical success (%)	Adverse events (%)
Siddiqui	2019	Retro	ETGBD	124	88	90	2
			EUS-GBD	102	94	80	5
			PTGBD	146	98	97	20
Jang	2012	RCT	EUS-GBD	30	97	100	7
			PTGBD	29	97	96	3
Itoi	2010	SR	PTGBA PTGBD ENGBD EGBS	122	93	83	0.8
				246	98	90	3.7
				194	81	75	3.6
				127	96	88	6.3
			EUS-GBD	12	100	100	16.7
Khan	2017	SR	ETGBD	647	83	93	10
			EUS-GBD	162	93	97	13
Mohan	2019	SR	ETGBD	1,223	83	88.1	9.6
			EUS-GBD	557	95.3	96.7	12.4
			PTGBD	13,351	98.7	89.3	15.1

*Retro* retrospective study; *RCT* randomized controlled trial; *SR* systematic review; *ETGBD* endoscopic transpapillary gallbladder drainage; *EUS-GBD* endoscopic ultrasound-guided gallbladder drainage; *PTGBD* percutaneous transhepatic gallbladder drainage; *PTGBA* percutaneous transhepatic gallbladder aspiration; *ENGBD* endoscopic naso-gallbladder drainage; *EGBS* endoscopic gallbladder stenting

Although EUS-GBD is a relatively new technique with reportedly high technical and clinical success rates (90–100%), institutions performing this procedure are limited [173, 211, 212, 214]. Recently, three new systematic reviews of EUS-GBD for acute cholecystitis using a lumen-apposing metal stent reported good results comparable to those of PTGBD in cases of acute cholecystitis [183, 217, 218]. At present, the EUS-GBD technique has not yet been generalized and hence, cannot be included in the recommendation.

## CQ3-(1)-5. Is endoscopic treatment recommended over surgery for the Mirizzi syndrome and confluence stone?

### Recommendations

- Surgical treatment is recommended for the Mirizzi syndrome.

(Recommendation: strong, 91% agreed, evidence level D).

- Endoscopic treatment with oral cholangioscopy at a well-equipped endoscopic institution is suggested for confluence stone.

(Recommendation: weak, 100% agreed, evidence level D).

### Commentary

The pathogenesis of Mirizzi syndrome is CBD stricture caused by compression of the common hepatic duct by stone and/or inflammation in the neck of the gallbladder. McSherry et al. classified it as type I without fistula and type II with gallbladder-bile duct fistulas. Csendes et al. classified McSherry type II into three subtypes: type II (fistula occupying 1/3 of the CBD), type III (fistula occupying 2/3 of the CBD), and type IV (fistula occupying the entire CBD width). MRCP is useful for diagnosis (44–82%) [219–221], and ERCP is performed not only for diagnosis but also for biliary drainage in cases of cholangitis and jaundice [222].

The standard treatment for Mirizzi syndrome is open surgery, including cholecystectomy and fistula closure. Temporary placement of a T-tube is carried out for small fistulas. The adverse event rate in patients with a fistula is reportedly high [219]. Laparoscopic surgery is difficult and has a higher rate of complications than open surgery [219, 223]. The conversion rate from laparoscopic surgery to open surgery is also high (8–76%) [219–221, 224, 225], with a complication rate of 12–19% [221, 224] including fatal cases [223, 224]. Laparoscopic treatment of Mirizzi syndrome should be performed only by skilled surgeons. [225, 226].

Endoscopic treatment is indicated for Mirizzi syndrome type II and confluence stones, which are located at the confluence of the cystic duct, common hepatic duct, and CBD. [227]. Peroral cholangioscopy with electrohydraulic lithotripsy (EHL) or YAG laser has shown a very high complete stone removal rate of 95% (92 of 97 cases) [228–230].

In Japan, endoscopic treatment is often performed using ESWL. Although endoscopic treatment of confluence stones is deemed to increase with the development and spread of devices, our recommendation has been weak (proposal) considering the local availability of this technique.

#### ***Common bile duct stones (see*** Fig. 6*).*

**Fig. 6:**
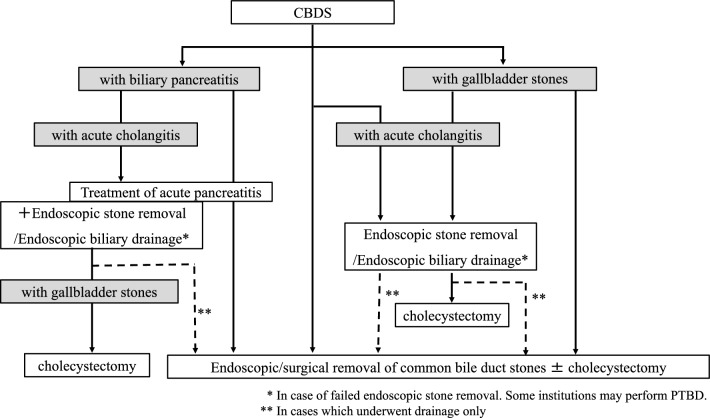
Flowchart for treatment of common bile duct stones

## BQ3-(2)-1. What kind of endoscopic treatments do we have for CBDS?

### Statements

– The standard treatment is endoscopic sphincterotomy (EST) with subsequent endoscopic stone extraction; however, endoscopic papillary balloon dilation (EPBD) is also applicable.
- In cases of endoscopically difficult stones, endoscopic papillary large balloon dilation (EPLBD), peroral cholangioscopy (POCS), percutaneous transhepatic cholangioscopy (PTCS), balloon endoscopy-assisted techniques, and EUS-guided procedures are also used.

These statements were made with reference to the following information and papers [231–233].

## BQ3-(2)-2. What are the surgical treatments for CBDS?

### Statement

- Surgical treatments for CBDS include "open surgery (cholecystectomy and choledocholithotomy),” "laparoscopic surgery (cholecystectomy and choledocholithotomy)," and "laparoscopic cholecystectomy and endoscopic removal of common bile duct stones."

This statement was made with reference to the following information and papers [10, 200, 234, 235].

## BQ3-(2)-3. What are the treatment options for CBDS associated with gallbladder stones?

### Statements

- There are various treatment options, including (1) surgical cholecystectomy after endoscopic bile duct stone removal and (2) simultaneous surgical cholecystectomy and bile duct stone removal (Table 2).

**Table 2:** Therapeutic Procedures for gallbladder stones and common bile duct stones

	Single session		Multi-session	
	Category	Procedure	Category	Procedure
Gallbladder stone	Surgery	Open surgical/ laparoscopic cholecystectomy	Combination	Endoscopic/percutaneous transhepatic gallbladder drainage → Open surgical/Laparoscopic cholecystectomy
Common bile duct stone without gallbladder stone	Endoscopy	Endoscopic removal of common bile duct stone	Endoscopy	Endoscopic biliary drainage → Endoscopic removal of common bile duct stone
	IR	Percutaneous removal of common bile duct stone	Combination	Percutaneous transhepatic biliary drainage → Endoscopic/Percutaneous removal of common bile duct stone
	Surgery	Open surgical/ laparoscopic common bile duct stone exploration with or without cholecystectomy	Combination	Percutaneous transhepatic biliary drainage → Open surgical/ laparoscopic common bile duct stone exploration with or without cholecystectomy
Gallbladder stone with common bile duct stone	Surgery	Open surgical/ laparoscopic cholecystectomy and common bile duct stone exploration	Combination	Endoscopic/percutaneous removal of common bile duct stone (before surgery) → Open surgical/ Laparoscopic cholecystectomy
	Combination	Laparoscopic cholecystectomy and intraoperative ERCP	Combination	Open surgical/ laparoscopic cholecystectomy → Endoscopic/percutaneous removal of common bile duct stone (after surgery)
			Combination	Endoscopic/Percutaneous transhepatic biliary drainage with or without Endoscopic/ percutaneous transhepatic gallbladder drainage → Cholecystectomy → Endoscopic/ percutaneous removal of common bile duct stone
			Combination	Endoscopic/Percutaneous transhepatic gallbladder drainage → Laparoscopic common bile duct stone exploration with or without cholecystectomy

*IR* interventional radiology, *ERCP* endoscopic retrograde cholangiopancreatography

This statement was made with reference to the following information and papers [236–243].

## BQ3-(2)-4. Is endoscopic CBDS removal plus surgical cholecystectomy (two-stage combined treatment) more beneficial than surgical CBDS removal plus cholecystectomy (one-stage surgical treatment) for CBDS complicated with gallbladder stones?

### Statement

- Endoscopic CBDS removal plus surgical cholecystectomy and surgical CBDS removal plus cholecystectomy are equally effective in complete ductal clearance, the rate of residual stones, mortality, and complications, except for longer hospital stay for endoscopic CBDS removal plus surgical cholecystectomy.
- In Japan, a two-stage combination treatment of endoscopic CBDS removal followed by surgical cholecystectomy is commonly performed.

These statements were made with reference to the following information and papers [10, 200, 244–248].

## FRQ3-(2)-1. Does surgical treatment for CBDS, which does not affect the function of the sphincter of Oddi, have a better long-term prognosis compared to that of endoscopic treatment?

### Statement

- A method of assessing the relationship between the prognosis and function of the sphincter of Oddi after treatment of CBDS has not been established. The long-term impact of surgical treatment of CBDS on the stone recurrence rate and quality of life of patients should be investigated.

## Commentary

The treatment of CBDSs with gallbladder calculi is generally a two-stage procedure consisting of endoscopic treatment (endoscopic CBDS removal) and laparoscopic cholecystectomy, although surgical one-stage treatment is also used. In Japan, 80% of the CBDSs are treated with the two-stage treatment [200].

One-stage treatment allows preservation of the functions of the sphincter of Oddi. The stone recurrence rate is lower with one-stage treatment than with two-stage treatment and is reportedly 3.5% at 10 years [249]. The correlation between the functionality of the sphincter of Oddi and stone recurrence rate is related to the type of CBDSs and their origin (i.e., primary or secondary) [250]. In particular, one-stage treatment for patients aged < 60 years with secondary or cholesterol stones has a low recurrence rate [251].

On the other hand, the relationship between primary bile duct stones and sphincter of Oddi function with respect to etiology, course, and prognosis is not well understood.

Currently, available methods for evaluating papillary function include endoscopic or constant-flow perfusion biliary pressure measurement, biliary excretion dynamics by biliary scintigraphy, and serial MRCP imaging [252–255]. The impact of surgical CBDS removal with preservation of papillary sphincter function on long-term prognosis, such as the stone recurrence rate and patients’ quality of life, should be clarified.

## FRQ3-(2)-2. Is laparoscopic surgery recommended over open surgery for one-stage surgical treatment of CBDS with gallbladder stones?

### Statement

- Supportive evidence for the recommendation of laparoscopic one-stage surgery for CBDS with gallbladder stones is insufficient, and further accumulation of data is necessary.

## Commentary

The one-stage surgical treatment of CBDSs with gallbladder stones consists of open surgery, which has been conventionally performed, and laparoscopic surgery. Laparoscopic surgery includes "laparoscopic cholecystectomy and choledocholithotomy," and laparoscopic cholecystectomy and intraoperative endoscopic removal of CBDS (rendezvous technique)". In recent years, reports of laparoscopic cholecystectomy and intraoperative endoscopic removal of CBDS have been increasing, mainly in Europe and the United States. In Japan, two-stage treatment is widely used, in which endoscopic removal of CBDS is performed first, followed by laparoscopic cholecystectomy. When one-stage surgical treatment is chosen, it is often difficult to perform endoscopic removal of CBDS for some reason.

According to a 2017 survey on endoscopic surgery by the Japan Society for Endoscopic Surgery, only 20 of the 353 centers (6%) performed laparoscopic choledocholithotomy in all cases, 196 centers (56%) performed laparoscopic choledocholithotomy in some cases, and 137 centers (39%) performed open choledocholithotomy in all cases, which is still a high percentage [200].

An RCT compared one-stage open versus one-stage laparoscopic choledocholithotomy in 256 patients with CBDS and found that there was no statistically significant difference in operative time, stone removal rate, or complication rate. However, laparoscopic choledocholithotomy was significantly better in terms of intraoperative bleeding, postoperative hospital stay, and wound infection rate [256]. As this single-center RCT is the only study to compare one-stage open versus one-stage laparoscopic choledocholithotomy, the evidence for the recommendation of the laparoscopic approach is currently insufficient.

One-stage laparoscopic cholecystectomy and choledocholithotomy should be performed safely, taking each institution's environment and surgeons' skills into account.

## CQ3-(2)-1. For patients with CBDS uncomplicated with gallbladder stones or post-cholecystectomy, is endoscopic treatment recommended over surgery?

### Recommendation

- Endoscopic treatment is recommended for CBDS that are uncomplicated by gallstones or with a history of cholecystectomy.

(Recommendation: strong, 100% agreed, evidence level C).

## Commentary

No RCTs or meta-analyses have compared endoscopy and surgery for CBDSs with uncomplicated gallbladder stones or previous cholecystectomy. Wang et al. [257] reported a retrospective study comparing endoscopic and laparoscopic treatments for CBDSs with previous cholecystectomy. The complete stone clearance rate after endoscopic treatment was significantly higher than that after laparoscopic surgery (97.7 vs. 87%; *p* = 0.003). The treatment time of endoscopy was significantly shorter than that of laparoscopy (52.0 ± 15.8 min vs. 102.9 ± 40.1 min, *p* < 0.001). The complication rate after treatment (endoscopy, 3.4%; laparoscopy, 11.1%, *p* = 0.15) and hospital stay (endoscopy, 5.5 ± 2.6 days; laparoscopy, 5.9 ± 2.3 min, *p* = 0.40) were not different.

Japan Biliary Association performed a multi-institutional prospective study on the treatment of CBDSs in 2013 [10]. Endoscopic treatment was most frequently performed for CBDSs without cholecystolithiasis (endoscopy, 71.2%; surgery, 7.6%).

Considering these results, endoscopy may be the first-choice treatment for CBDSs uncomplicated by gallbladder stones or after cholecystectomy.

## CQ3-(2)-2. For asymptomatic CBDS, is stone removal recommended over follow-up without treatment?

### Recommendation

- Stone removal is proposed for asymptomatic CBDS because of the risk of cholangitis and other complications.

(Recommendation: weak, 82% agreed, evidence level C).

## Commentary

Stone removal is generally performed for CBDSs regardless of the presence or absence of symptoms because of the risk of severe cholangitis and other complications [258]. However, the natural history of asymptomatic CBDSs remains unclear. Previous reports have shown that asymptomatic CBDSs are detected in 10–20% of the patients undergoing cholecystectomy [259–261]. Kim et al. reported the results of endoscopic treatment for CBDSs according to the presence or absence of symptoms [262] and reported that the incidence of pancreatitis after ERCP was significantly higher in patients with asymptomatic CBDSs (12.5 vs. 3.9%, *p* = 0.045). Hakuta et al. [263] reported an observational study of 191 asymptomatic cases of CBDSs: 114 cases in the wait-and-see group and 77 in the intervention group. The cumulative incidence of biliary complications was 6.1% at 1 year, 11% at 3 years, and 17% at 5 years, with no differences between the groups (*p* = 0.55). Procedure-related adverse events were observed in 22 patients (19%), including 4 (5.2%) with severe pancreatitis in the wait-and-see group and 25 (32%) in the early endoscopic removal group. They concluded that a wait-and-see strategy is an option, considering the incidence of procedure-related complications of endoscopic intervention for asymptomatic patients. At present, asymptomatic CBDSs are often treated endoscopically; however, the benefits and disadvantages of endoscopic treatment should be carefully evaluated, especially in the elderly, patients with poor activity of daily living (ADL), and those with serious underlying diseases.

## CQ3-(2)-3. Is single-session stone removal recommended in cases of bile duct stones with acute cholangitis?

### Recommendation

- Single-session stone removal can be safely performed in patients with CBDSs and acute cholangitis. Bile duct drainage only in the first session and scheduled stone removal are suggested depending on the patient's condition.

(Recommendation: strong, 100% agreed, evidence level C).

## Commentary

For patients with common duct stones associated with acute cholangitis, there are two treatment options: (1) removal of stones in a single session and (2) performing biliary drainage only in the first session and waiting for the improvement of cholangitis before performing stone removal. In cases of severe acute cholangitis, short-term biliary drainage with stent placement (endoscopic biliary stenting (EBS) or endoscopic nasobiliary drainage (ENBD)) is recommended [264]. It is also safer to treat cholangitis with EBS alone without EST and to perform scheduled stone removal after the resolution of acute cholangitis, although the length of hospital stay is prolonged [265]. When complete stone removal is achieved, biliary drainage, such as EBS or ENBD, is not always necessary [266]. Eto et al. reported excellent results for single-session stone removal in 50 patients [267]. However, it should be noted that this study did not include patients with severe cholangitis or poor general condition. In addition, patients with a bleeding tendency or those undergoing antithrombotic treatment may be at risk of bleeding during/following endoscopic procedures, such as EST. The size and number of stones can make a single-session treatment difficult. Therefore, it is important to carefully evaluate the patient's general condition, the number and diameter of stones, and the severity grade of cholangitis according to Tokyo Guidelines (TG) 18 before treatment. If the equipment in the facility including the skill of endoscopists is not sufficient to perform the procedure safely, transfer of the patient to a specialized center should be considered [142]

## CQ3-(2)-4. Is endoscopic treatment recommended over conservative treatment for biliary pancreatitis?

### Recommendation

- Early endoscopic treatment is suggested for biliary pancreatitis associated with acute cholangitis.

(Recommendation: strong, 100% agreed, evidence level C).

## Commentary

Multiple meta-analyses have compared early ERCP with conservative treatment for biliary pancreatitis [268–272]. Since ERCP is necessary for the management of acute cholangitis, the inclusion of patients with or without acute cholangitis has a great impact on the results of the meta-analyses. In the analyses that excluded patients with biliary pancreatitis associated with acute cholangitis [269, 270], early ERCP for biliary pancreatitis did not show any difference in morbidity and mortality rates compared to conservative management, regardless of the severity of pancreatitis. According to the analysis by Tse in 2012 [271], early ERCP significantly reduced mortality rates (RR, 0.20; 95% CI 0.06–0.68) and both local (RR, 0.45; 95% CI 0.20–0.99) and the general (RR, 0.37; 95% CI 0.18–0.78) adverse event rates if only RCTs including patients with biliary pancreatitis associated with acute cholangitis were analyzed. However, early ERCP showed a tendency to increase the mortality rate (RR, 1.91; 95% CI 0.85–4.30) and the local (RR, 1.15; 95% CI 0.69–1.92) and general (RR, 1.02; 95% CI 0.44–2.36) adverse event rates when only RCTs that excluded patients with acute cholangitis were analyzed. Based on these results, it is not recommended to perform early ERCP routinely in all patients with biliary pancreatitis. Early endoscopic treatment is suggested for cases of biliary pancreatitis associated with definite/suspected acute cholangitis. The diagnosis of acute cholangitis should be based on cholestatic findings on blood examinations and imaging findings since inflammatory findings can be caused by pancreatitis only. When endoscopic treatment of biliary pancreatitis associated with acute cholangitis is difficult, PTBD should be considered to manage acute cholangitis.

## CQ3-(2)-5. Is EPLBD more recommended than EST for large or multiple bile duct stones?

### Recommendation

- EPLBD is recommended for large or multiple bile duct stones.

(Recommendation: strong, 91% agreed, evidence level A).

## Commentary

EPLBD, first reported by Ersoz et al. [273] in 2003, is a procedure to dilate the duodenal papilla using a large-diameter balloon of 12 mm or more in diameter with or without EST [274]. EPLBD is used to manage difficult bile duct stones, such as large or multiple stones because the biliary orifice is opened sufficiently after dilation using a large-diameter balloon. Several meta-analyses have evaluated the efficacy and safety of EPLBD in the management of bile duct stones [275–280]. A recent meta-analysis, including 9 RCTs and 9 non-RCTs, compared EPLBD with EST to EST alone for large (≥ 10 mm) or multiple bile duct stones [279]. The results showed that EPLBD with EST was significantly superior in both the total stone removal rate and the rate of stone removal in the first ERCP session, with less frequent use of mechanical lithotripsy. With regard to safety, EPLBD with EST showed a lower rate of overall early adverse events than EST alone. The incidence of significant bleeding was lower in the EPLBD with EST group than in the EST group; however, no difference was observed in the incidence of pancreatitis, acute cholangitis, or perforation between the groups. According to the Japan Gastroenterological Endoscopy Society guidelines for EPLBD, EPLBD is contraindicated in cases of distal bile duct stricture or nondilated bile duct because of the higher risk of perforation [232]. A meta-analysis evaluated the significance of EST before EPLBD and reported that EPLBD with EST showed similar efficacy and safety as EPLBD without EST [280]. Some studies comparing the long-term results between EPLBD and EST for large/multiple bile duct stones showed no significant differences in the incidence of long-term adverse events between the procedures, although further evaluation of safety is required because these reports are retrospective studies with insufficient follow-up periods [281, 282].

## CQ3-(2)-6. Is endoscopic treatment recommended for giant CBDS?

### Recommendation

- For giant stones, endoscopic treatment with ESWL or POCS is performed at specialized centers; however, there is little evidence to support recommending its use.

(Recommendation: none, 91% agreed, evidence level D).

## Commentary

EPLBD is recommended for large stones (see CQ3-(2)-5), but giant stones > 3 cm are difficult to treat endoscopically, and ESWL has been used as an adjuvant treatment. It has been reported that a combination of ESWL with endoscopy achieves stone removal in 80–90% of the cases of giant bile duct stones [283]. Recently, with the advance of peroral cholangioscopy (POCS), endoscopic treatment of giant stones has been aggressively performed. In a meta-analysis of POCS for difficult-to-treat stones [284], the stone removal rate was 94.3%, of which 71.1% were successful in one session, and the complication rate was low (6.1%). Technically, lithotripsy under POCS is performed using laser or electrohydraulic lithotripsy (EHL); and the stone removal rate with the use of laser and that using EHL is reportedly 85–98% and 64–97%, respectively, while the removal rate using EHL is 64–97% [285].

In contrast, the stone removal rates of LCBDE and laparotomy in cases of unsuccessful endoscopic stone removal have been reported to be 95.2% and 95%, respectively [286].

The importance of POCS has been increasing with technological innovations and advances in equipment, and endoscopic treatment of giant stones, which was not previously indicated, is now being performed at specialized centers.

In addition to surgical and endoscopic treatment, stone removal by PTCS after PTBD is also useful [287].

## CQ3-(2)-7. In the case of CBDS with altered anatomy after upper gastrointestinal surgery, what kind of treatment should be selected for stone removal?

### Recommendation

- Balloon endoscopy is proposed for CBDS removal by experienced surgeons in patients with post-surgically altered upper gastrointestinal anatomy.
- (Recommendation: weak, 100% agreed, evidence level C).

## Commentary

The treatment methods for CBDS in patients with altered anatomy after upper gastrointestinal surgery, such as Roux-en-Y (R-Y) or Billroth II (B-II), include endoscopic, percutaneous, and surgical stone removal. Each treatment has individual advantages and disadvantages [288]. However, no report has compared these three methods. Successful endoscopic approach to the papilla and treatment success in B-II reconstruction cases using a conventional endoscope are 72–97% and 49–92%, respectively [289–293]; however, the results in R-Y cases are not satisfactory. The development of double-balloon and single-balloon endoscopes has made it easy to reach the duodenal papilla in cases of patients with a surgically altered anatomy [294–296], and these scopes are now widely used in endoscopic biliopancreatic interventions in daily clinical practice. Satisfactory results of ERCP in R-Y cases using a balloon endoscope have been reported as 92.6–97% for successful approaches to the papilla, 58–95.6% for successful cannulation of the bile duct, with an adverse event rate of 7.3–10.3% [294, 297–299]. However, this procedure remains challenging and entails a potential risk of severe adverse events. Therefore, balloon endoscopy-assisted ERCP should be performed by experienced endoscopists at specialized facilities [300, 301]. Recently, there have been several reports on the usefulness of interventional EUS for such patients, which is expected to be an alternative treatment method in cases of failed balloon endoscopy-assisted ERCP [302, 303].

## BQ3-(2)-5. Is percutaneous transhepatic biliary drainage useful for acute cholangitis with CBDS when endoscopic transpapillary drainage is difficult?

### Statement

- Percutaneous transhepatic biliary drainage is useful in difficult cases for endoscopic transpapillary drainage, such as in patients with postoperative bowel reconstruction or unsuccessful transpapillary cannulation. EUS-BD has also emerged as a new treatment option.

This statement was made with reference to the following information and papers [304, 305].

## BQ-3-(2)-6. What kind of patients with CBDS are good indications for EPBD?

### Statement

- Patients with bleeding diathesis are good indications for EPBD.

These statements were made with reference to the following information and papers [306–314].

## CQ3-(2)-8 Is endoscopic treatment recommended for CBDS in patients receiving antithrombotic therapy?

### Statement:

- Endoscopic treatment is recommended for CBDS in patients receiving antithrombotic therapy, as described in the relevant guidelines.

(Recommendation: weak, 82% agreed, evidence level C).

## Commentary

According to the Japanese guidelines [315, 316], patients with CBDS and acute cholangitis who also have coagulopathy, and those receiving antithrombotic therapy should initially undergo ENBD or EBS, that is, drainage alone, to improve cholangitis along with coagulopathy, and then undergo CBDS removal via EST, which should be performed after the withdrawal of the antithrombotic agent, for safety [317]. Some patients may undergo ENBD or EBS alone depending on their condition. EST and EPLBD should be performed in accordance with the corresponding guidelines [231, 232]. In patients at high risk of thromboembolism, who develop acute cholangitis, if such patients are receiving aspirin alone, EST or EPLBD without drug withdrawal can be acceptable after consultations with specialists in antithrombotic therapy. Patients receiving thienopyridine derivatives other than aspirin have been reported to have a higher risk of accidental bleeding; therefore, these drugs should be substituted with aspirin or cilostazol [231, 232]. When only warfarin is used as an anticoagulant, EST or EPLBD can be performed with warfarin therapy if the international normalized ratio is within the therapeutic range. EST or EPLBD can be performed in patients with nonvalvular atrial fibrillation after temporarily switching from warfarin therapy to direct oral anticoagulant (DOAC) therapy [316]. DOAC monotherapy should be continued until the day before EST or EPLBD is performed and resumed on the 1^st^ postoperative day.

EPBD is a good alternative to EST for the patient groups discussed here. The incidence of accidental procedure-related bleeding is significantly lower in patients undergoing EPBD than in those undergoing EST as reported in a meta-analysis [1].

## CQ3-(2)-9 Is endoscopic stone removal recommended over permanent biliary stenting for CBDS in the elderly and in patients with serious underlying comorbidities?

### Statement

- Endoscopic stone removal is recommended over permanent biliary stenting for CBDS in the elderly and in patients with serious underlying comorbidities. Permanent biliary stenting should be considered only in patients with a poor prognosis.
- (Recommendation: weak, 100% agreed, evidence level C).

## Commentary

The mainstay of endoscopic treatment for CBDS is complete stone removal after the duodenal papillary intervention. However, the elderly and patients with serious underlying diseases may not be able to endure long procedure time for endoscopic treatment of stones and are at risk of developing procedural complications.

Three retrospective studies and one RCT compared endoscopic stone removal and permanent stent placement for CBDS in the elderly and in patients with serious underlying diseases [318–320]. In all studies, no significant differences were observed in the incidence of short-term procedural complications between the two groups. However, the incidence of long-term biliary-related complications was significantly higher in the permanent biliary stenting group than in the endoscopic stone removal group, and the onset of long-term complications was earlier in patients undergoing permanent biliary stenting.

These findings suggest that endoscopic stone removal for CBDS is preferred over permanent biliary stenting, even in the elderly and patients with serious underlying diseases. Permanent biliary stenting should be considered only in patients with poor prognosis. After permanent stent placement, it is necessary to identify the signs of acute cholangitis, such as fever, jaundice, and abdominal pain, for the early detection and treatment of biliary stent-related complications.

## CQ3-(2)-10. Is a balloon catheter more useful than a basket catheter in endoscopic stone removal for CBDS?

### Recommendation

- Balloon catheters are as useful as basket catheters in endoscopic stone removal for CBDS and both are recommended.

(Recommendation: strong, 100% agreed, evidence level B).

## Commentary

Endoscopic extraction of CBDS is generally performed using a balloon or basket catheter following EST. The choice between the two devices depends largely on the preferences of the operator and the institution. Basket catheters are generally used first in Japan and Europe [321, 322], while balloons are overwhelmingly used in the United States because of concerns about basket impaction [323].

Ishiwatari et al. [324] compared the complete stone removal rate of a balloon and a basket in an RCT on 172 patients with CBDS with a diameter < 10 mm (bile duct diameter < 15 mm) and found that the rate was significantly higher in the balloon group than in the basket group (92.3 vs. 80.0%, *p* = 0.037). Ozawa et al. [325] also compared the successful complete stone removal rate within 10 min in a randomized noninferiority study of 184 patients (CBDS diameter < 11 mm)The success rates were comparable (83.9% in the balloon group and 81.3% in the basket group), while the incidence of adverse events was also similar (11.8% and 6.6%). The reasons for failure in the balloon group were that the balloon passed through the duct beside the stone or the operator lost sight of the stone during the procedure; moreover, small stones tend to lodge at the pocket-like lower-end corner of the bile duct terminal. On the other hand, in the basket group, when the stones were very small, they slipped between the wires and were difficult to grasp.

As the results of the two RCTs have led to different conclusions, additional studies are needed. The recently published guidelines of the European Society of Gastrointestinal Endoscopy have concluded that the efficacy and safety of the balloon and basket are equivalent [326].

## CQ3-(2)-11. Is cholecystectomy recommended over follow-up after endoscopic choledocholithotomy?

### Recommendation

- For CBDS with gallstones, cholecystectomy is recommended after endoscopic choledocholithotomy.

(Recommendation: strong, 91% agreed, evidence level B).

- For CBDS without gallstones, there is a lack of evidence to recommend additional cholecystectomy after endoscopic choledocholithotomy.

(Recommendation: none, 100% agreed, evidence level C).

## Commentary

In a meta-analysis of five RCTs that compared cholecystectomy and follow-up in patients with gallstones who underwent endoscopic choledocholithotomy, there were significantly more deaths in the follow-up group, a higher incidence of pain due to biliary tract, a higher incidence of recurrent jaundice and cholangitis, and a higher incidence of repeat cholangiography, such as ERCP [243, 327–330]. Furthermore, in the follow-up group, 35% of the patients underwent cholecystectomy during the follow-up period [331].

In a retrospective study of a large number of cases using the Korean National Health Insurance database, among the 16,910 patients with gallstones who were treated endoscopically for CBDS, the recurrence rate of CBDS was significantly lower among patients who underwent cholecystectomy, which was 8% (920 in 11,617 patients), compared to patients who did not undergo cholecystectomy, which was 15% (773 in 5293 patients) (*p* < 0.0001) [332]. These results indicate that there is a consensus that cholecystectomy is more effective than follow-up after endoscopic choledocholithotomy.

In contrast, a long-term retrospective study of patients with CBDS who were treated with endoscopic choledocholithotomy, including many cases without gallstones, showed no difference in the recurrence rate of CBDS between the follow-up and cholecystectomy groups at a median follow-up period of 5 years or longer (15 vs. 19%, *p* = 0.295) [333]. These results suggest that prophylactic cholecystectomy is not necessary after endoscopic choledocholithotomy in patients without gallstones, although further validation is necessary.

## FRQ3-(2)-3. Is oral administration of a choleretic after removal of CBDS more useful than no treatment?

### Statement

- The efficacy of ursodeoxycholic acid (UDCA) after CBDS removal in preventing recurrence has not been proven.

### Commentary

There are few reports on the outcomes of UDCA administration after the treatment of CBDS [334]. Yamamoto et al. reported in an RCT that the recurrence rate after the removal of CBDS was 6.6% in the UDCA group and 18.6% in the non-UDCA group, although the study included only a small number of patients [335]. In their multivariate analysis, only the UDCA group showed a significant difference, with a hazard ratio of 5.032 (95% CI 1.011–39.75, *p* = 0.048), indicating that the UDCA was effective against recurrent CBDS. Conversely, there have been reports of frequent recurrence of CBDS after the administration of UDCA [336, 337]. The acidic pH of bile is thought to be the cause of stone formation, as UDCA tends to precipitate more easily. In any case, there are no meta-analyses or large-scale randomized trials that provide clear evidence at present, and the choice of treatment should be determined on a case-by-case basis.

#### ***Hepatolithiasis ***(see Fig. 7a, b).

**Fig. 7:**
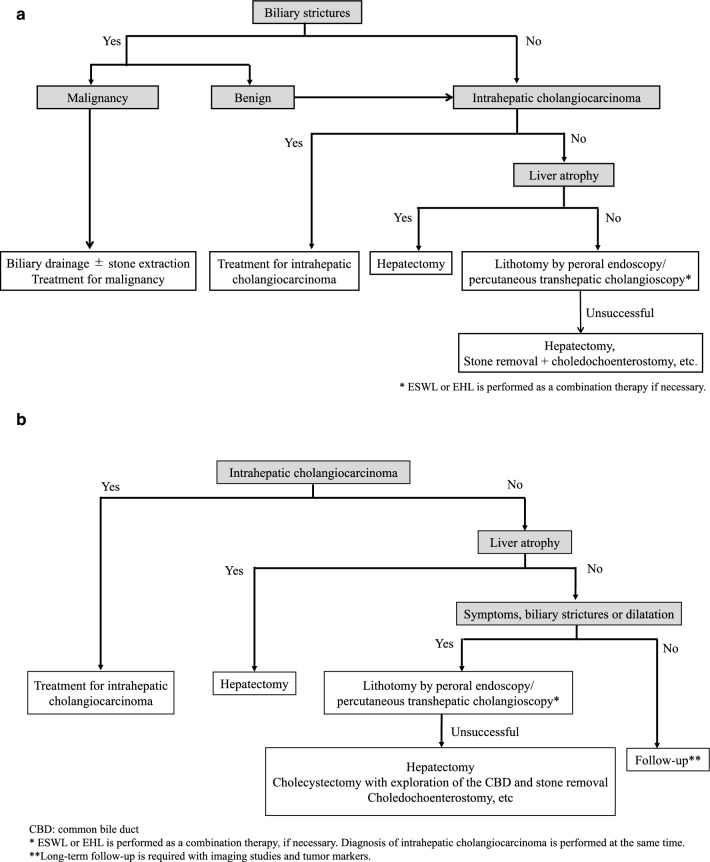
a Flowchart for treatment of hepatolithiasis with previous choledochoenterostomy. b Flowchart for treatment of hepatolithiasis without previous choledochoenterostomy

## BQ3-(3)-1. What are the procedures for treating hepatolithiasis?

### Statements

- Hepatectomy, choledochoenterostomy, choledochotomy, stone removal with or without T-tube drainage, and plasty of the duodenal papilla are surgical treatments for hepatolithiasis.
- ESWL, percutaneous transhepatic cholangioscopic lithotripsy, EUS-guided hepaticogastrostomy, endoscopic retrograde cholangiography (ERC), balloon-assisted ERC with stone extraction, and POCS with stone extraction have also been performed as nonsurgical treatments.

These statements were made with reference to the following information and papers [11, 161].

## BQ3-(3)-2. What are the indications for hepatectomy in cases of hepatolithiasis?

### Statements

- Hepatectomy is indicated in patients with concomitant intrahepatic cholangiocarcinoma and/or liver atrophy.
- Hepatectomy is considered in patients with failed nonsurgical treatments.

These statements were made with reference to the following information and papers [1, 11, 101, 111, 338, 339].

## CQ3-(3)-1. Is observation recommended for asymptomatic hepatolithiasis?

### Statements

- For asymptomatic hepatolithiasis without concomitant cholangiocarcinoma*, liver atrophy*, biliary stricture**, or biliary dilatation**, follow-up without surgery is suggested.

(*:Recommendation: weak, 100% agreed, evidence level C).

(**:Recommendation: weak, 82% agreed, evidence level D).

## Commentary

Few studies have reported the long-term outcomes of asymptomatic hepatolithiasis. In asymptomatic hepatolithiasis, liver atrophy occurs less frequently than in symptomatic hepatolithiasis [340]. Furthermore, after hepatectomy for hepatolithiasis, cholangitis occurs more frequently in patients with remnant biliary strictures than in patients without biliary strictures [341]. Considering these results, follow-up is appropriate for asymptomatic hepatolithiasis without concomitant intrahepatic cholangiocarcinoma, liver atrophy, biliary strictures, or biliary dilatations. During follow-up, imaging studies and tests for tumor markers are required for the early detection of intrahepatic cholangiocarcinoma.

## BQ3-(3)-3. Is ESWL useful for the treatment of hepatolithiasis?

### Statement

- The complete stone clearance rate of ESWL is not high. However, ESWL is useful when used in combination with endoscopic treatment.

This statement was made with reference to the following information and papers [1, 11, 342–349].

## BQ3-(3)-4. Is peroral endoscopic treatment useful compared with percutaneous transhepatic cholangioscopic lithotripsy in cases of hepatolithiasis?

### Statement

- The treatment outcomes of peroral endoscopic procedures, including ERC, balloon-assisted ERC, and peroral cholangioscopy on stone extraction, are similar to those of percutaneous transhepatic cholangioscopic lithotripsy.

This statement was made with reference to the following information and papers [11, 98, 113, 161, 285, 350–352].

## Prognosis and adverse events

### BQ 4–1. What are the long-term complications after cholecystectomy for gallbladder stones?

#### Statement

- Residual or new stones in the bile duct or cystic duct, intraperitoneal migration of gallstones, insufficiency of the papillary sphincter, biliary tract injury, incisional hernia, and surgically induced changes in bile acid metabolism have been reported.

This statement was made with reference to the following information and papers [353–355].

## BQ4-2. Does cholecystectomy impair digestive and absorptive function?

### Statement

- There is no clear evidence that cholecystectomy impairs digestive and absorptive function.

This statement was made with reference to the following information and papers [356–362].

## BQ4-3. Are residual stones a risk factor for acute cholangitis and liver abscess after removal of CBD or intrahepatic stones?

### Statement

- Although it is difficult to determine whether the stones are residual or recurrent, they might be risk factors for acute cholangitis and liver abscesses.

This statement was made with reference to the following information and papers [332, 363–373].

## BQ4-4. What are the long-term complications after treatment of CBDSs?

### Statement

- Long-term complications included recurrent bile duct stones, acute cholangitis, and acute cholecystitis.

This statement was made with reference to the following information and papers [273, 314, 332, 363, 364, 366–380]

## BQ4-5. What are the long-term complications after treatment of hepatolithiasis?

### Statement

- Stone recurrence was the most common complication, followed by acute cholangitis, liver abscess, and intrahepatic bile duct cancer.

This statement was made with reference to the following information and papers [93, 94, 98, 107, 381–388].

## Appendix 1

The members of the Guidelines Committee who created and evaluated the Japanese Society of Gastroenterology, ‘‘Evidence-based clinical practice guidelines for cholelithiasis’’ are listed below.

Creation Committee: Chair: Naotaka Fujita (Miyagi Medical Check-up Plaza). Vice-chair: Itaru Endo (Department of Gastroenterological Surgery, Yokohama City University), Ichiro Yasuda (Third Department of Internal Medicine, University of Toyama, Toyama). Members: Hiroyuki Isayama (Department of Gastroenterology, Graduate School of Medicine, Juntendo University), Takuji Iwashita (First Department of Internal Medicine, Gifu University Hospital) Toshiharu Ueki (Department of Gastroenterology, Fukuoka University Chikushi Hospital), Kenichiro Uemura (Department of Surgery, Graduate School of Biomedical & Health Sciences, Hiroshima University), Akiko Umezawa (Minimally Invasive Surgery Center, Yotsuya Medical Cube), Akio Katanuma (Center for Gastroenterology, Teine-Keijinkai Hospital), Yu Katayose (Division of Hepato-biliary-pancreatic Surgery, Tohoku Medical and Pharmaceutical University), Yutaka Suzuki (Department of General and Gastroenterological Surgery, Kyorin University School of Medicine).

Evaluation Committee: Chair: Michiaki Unno (Department of Surgery, Tohoku University Graduate School of Medicine). Vice-chair: Kazuo Inui (Department of Gastroenterology Yamashita Hospital). Members: Junichi Shoda (Division of Medical Sciences, Faculty of Medicine, University of Tsukuba), Toshio Tsuyuguchi (Department of Gastroenterology, Chiba Prefectural Sawara Hospital), Toshifumi Wakai (Division of Digestive and General Surgery, Niigata University Graduate School of Medical and Dental Sciences).

The Japanese Society of Gastroenterology: President: Satoshi Mochida (Department of Gastroenterology and Hepatology, Faculty of Medicine, Saitama Medical University). Past President: Kazuhiko Koike (Kanto Central Hospital). Director Responsible: Yoshifumi Takeyama (Department of Surgery, Kindai University Faculty of Medicine), Takao Itoi (Department of Gastroenterology and Hepatology, Tokyo Medical University).
